# Glaucoma neurodegeneration: targeted modulation of intraocular immune pathways – a review of novel therapeutic advances

**DOI:** 10.3389/fimmu.2026.1932850

**Published:** 2026-10-15

**Authors:** Yunyun Sun, Keyun Xue, Congqin Wen, Xinyan Ma, Xiaojie Hu

**Affiliations:** Wenzhou People’s Hospital, Wenzhou, China

**Keywords:** glaucoma, microglia, neuroimmunology, neuroinflammation, neuroprotection, targeted therapy

## Abstract

Glaucoma is the leading global cause of irreversible blindness, with its pathogenesis extending far beyond elevated intraocular pressure. Dysregulation of the intraocular immune system plays a central role in the progressive death of retinal ganglion cells. This review systematically examines the research progress on key intraocular immune pathways in glaucomatous neurodegeneration, with a primary focus on immune-inflammatory responses mediated by microglia, astrocytes, and T cells. It delves into the molecular mechanisms of various immunoregulatory networks, including the TLR4/nuclear factor kappa B (TLR4/NF-κB), Janus kinase/signal transducer and activator of transcription (JAK/STAT), mitogen-activated protein kinase (MAPK), complement, and T-cell receptor signaling pathways. The review analyzes potential therapeutic strategies targeting these pathways. By integrating the latest evidence from basic research and translational medicine, this article aims to provide a theoretical foundation and directional guidance for developing neuroprotective immunomodulatory therapies that go beyond intraocular pressure reduction.

## 1 Introduction

Glaucoma, a leading cause of irreversible blindness worldwide, is fundamentally a neurodegenerative disease characterized by the progressive loss of retinal ganglion cells (RGCs) and their axons, culminating in optic nerve damage and visual field defects (1, 2). For decades, the primary and often sole focus of clinical management has been the reduction of intraocular pressure (IOP), the most significant modifiable risk factor. However, a critical clinical paradox persists: a substantial proportion of patients, including those with normal-tension glaucoma (NTG), continue to experience disease progression despite effective IOP control (2, 3). This observation underscores the insufficiency of IOP-centric models and compellingly indicates the involvement of IOP-independent mechanisms in driving glaucomatous neurodegeneration. In recent years, increasing evidence from human tissues and experimental models has supported the involvement of dysregulated immune responses and chronic, low-grade neuroinflammation in glaucoma (4–7).

The eye, while an immune-privileged site, possesses a sophisticated resident immune system primarily composed of glial cells. In the retina and optic nerve head, microglia and astrocytes are now recognized as central orchestrators of the neuroinflammatory cascade in glaucoma (4–7). In response to primary stressors such as elevated IOP, ischemia, aging, or metabolic stress, these normally quiescent sentinel cells become activated (5, 8–10). This activation represents an initial, adaptive attempt to restore tissue homeostasis, but when the insult is chronic or excessive, it transitions into a maladaptive, sustained reactive state (4, 6, 7, 11). Activated microglia undergo morphological changes, proliferate, and release a plethora of pro-inflammatory cytokines (e.g., TNF-α, IL-1β, IL-6), chemokines, reactive oxygen species (ROS), and other cytotoxic mediators that may contribute to neuronal injury (6, 12). Similarly, reactive astrocytes (astrogliosis) contribute to neurotoxicity by altering the extracellular matrix, disrupting the blood-retinal barrier (BRB), and releasing inflammatory mediators (7). This glia-driven inflammatory milieu creates a hostile environment that directly damages RGC somas, synapses, and axons, thereby exacerbating neurodegeneration (4- (7). Notably, the interplay between microglia and macroglia (astrocytes and Müller cells) is not merely additive but synergistic, forming a complex cellular network that amplifies the inflammatory response and dictates the neurodegenerative outcome (5, 13).

Beyond innate immunity, accumulating evidence suggests that the adaptive immune system also contributes to glaucoma pathogenesis, highlighting potential overlap between neurodegenerative and autoimmune mechanisms (11, 14, 15). Studies have revealed a link between elevated IOP and T cell-mediated autoimmune responses, particularly those directed against stress-induced antigens like heat shock proteins (HSPs) (14). In experimental models, immunization with optic nerve antigens can induce glaucoma-like damage independent of IOP elevation, providing direct evidence for immune-mediated neurodegeneration (15). Furthermore, clinical data show that patients with autoimmune conditions such as rheumatoid arthritis have a significantly higher risk of developing primary open-angle glaucoma, suggesting shared immune-mediated pathophysiological pathways (16). The involvement of adaptive immunity is further supported by findings that clonally expanded HSP-specific T cells contribute to neurodegeneration via specific signaling pathways like mechanistic target of rapamycin complex 1 (mTORC1) (17). This adaptive response may be primed by the initial innate glial activation and the release of damage-associated molecular patterns (DAMPs) from stressed or dying neurons, leading to a vicious cycle of inflammation and neuronal loss (11, 15, 18). The breakdown of the BRB, observed in both acute and chronic models, facilitates the infiltration of peripheral immune cells, including macrophages and lymphocytes, which further augment the local inflammatory damage (11, 19).

The molecular underpinnings of this immune dysregulation are multifaceted and involve several key signaling pathways. Oxidative stress, arising from mitochondrial dysfunction and age-related metabolic decline, is a critical upstream driver that both triggers and is exacerbated by neuroinflammation (8, 20, 21). Damaged mitochondria release mitochondrial DNA (mtDNA), which acts as a DAMP to activate intracellular sensors like the NOD-, LRR-, and pyrin domain-containing protein 3 (NLRP3) inflammasome and the cyclic GMP–AMP synthase–stimulator of interferon genes (cGAS–STING) pathway in microglia, leading to the production of potent pro-inflammatory cytokines such as IL-1β and type I interferons (22–24). The complement system, another arm of innate immunity, is also activated in glaucoma. Studies in animal models demonstrate that complement components, including the anaphylatoxin receptor C3aR1 and the alternative pathway, promote microglial reactivity, neuroinflammation, and subsequent RGC loss (25, 26). Furthermore, pattern recognition receptors such as Toll-like receptor 4 (TLR4) are implicated in sensing endogenous DAMPs (e.g., HMGB1, S100 proteins) and transducing signals through NF-κB and MAPK pathways, resulting in the sustained production of inflammatory mediators (27). The convergence of these pathways on glial cells establishes a state of chronic neuroinflammation that is now considered a hallmark of glaucomatous neurodegeneration and a promising frontier for therapeutic intervention beyond IOP reduction (4, 5, 11).

## 2 The core role of microglia-mediated innate immune responses in glaucoma

### 2.1 Dynamic regulation of microglial activation and phenotypic switching

In glaucoma models, elevated IOP or optic nerve injury rapidly activates retinal microglia, transforming their morphology from a resting, ramified state to an activated, amoeboid shape (28). This activation is an early event, occurring before significant RGC death, and is characterized by changes in cell density and immunoreactivity (28, 29). The activation of microglia is multifactorial, driven by biomechanical, metabolic, and inflammatory signals, and these cells play a central role in regulating the retinal immune microenvironment (29) (30–32). Once activated, microglia exhibit dynamic phenotypic changes rather than a simple transition between discrete functional categories. Although the classical M1/M2 framework has been widely used to describe pro-inflammatory and anti-inflammatory microglial states, this binary classification is increasingly recognized as an oversimplification that does not fully capture the heterogeneous and context-dependent nature of microglial responses (33). Recent studies using single-cell transcriptomics have revealed diverse microglial states, including disease-associated microglia (DAM) and other transcriptionally defined phenotypes, which may coexist and dynamically evolve during disease progression (32). Therefore, when M1/M2-related terms are used in this review, they are intended only as a simplified description of broad inflammatory or reparative tendencies reported in earlier glaucoma studies, rather than as evidence for discrete or stable microglial subtypes. In the early stages of injury, microglia may exhibit M2-like characteristics associated with neuroprotective functions (34). However, chronic glaucomatous stress may promote the accumulation of pro-inflammatory microglial states characterized by increased expression of cytokines such as tumor necrosis factor alpha (TNF-α), interleukin-1 beta (IL-1β), and interleukin-6 (IL-6), contributing to neuroinflammation and RGC injury (31, 32, 35). This phenotypic conversion is not static but is dynamically regulated by various factors over time. For instance, interleukin-17A (IL-17A) has been identified as a key modulator in this process. In experimental glaucoma, IL-17A is significantly upregulated in the retina and promotes microglial activation and the release of pro-inflammatory cytokines (35). Furthermore, IL-17A dynamically regulates the phenotypic conversion of activated microglia, favoring early-stage neuroprotective transcriptional programs and promoting late-stage pro-inflammatory microglial states in glaucomatous retinas (35). Mechanistically, IL-17A-induced overactivation of microglia is mediated through the p38 MAPK signaling pathway, and blocking this pathway alleviates the effects (35). Consequently, suppression or knockdown of IL-17A has been shown to significantly diminish retinal inflammation, improve RGC survival, and enhance axonal quality and visual function in experimental glaucoma, highlighting its potential as a therapeutic target (35). Other interventions, such as the administration of irisin or Caveolin-1 (Cav-1), have also been demonstrated to promote a shift of microglial responses and enhance reparative or homeostatic features, thereby attenuating neuroinflammation and protecting RGCs (34, 36). This complex, time-dependent regulation of microglial activation and phenotype underscores their dual role in both neurotoxic injury and neuroprotective responses within the glaucomatous retina (30, 37). Therefore, while M1/M2 terminology provides a convenient framework for describing functional polarization in experimental glaucoma studies, future investigations integrating single-cell transcriptomics and spatial profiling will be essential for defining the complex microglial states involved in glaucomatous neurodegeneration.

### 2.2 Activation mechanisms of key innate immune signaling pathways

The progression of glaucoma involves the activation of multiple innate immune signaling pathways across retinal immune and glial cell populations, which collectively contribute to neuroinflammatory injury. The TLR4/NF-κB pathway is a central innate immune signaling axis that regulates inflammatory responses across multiple cell types, including microglia, astrocytes, and infiltrating immune cells. In chronic high IOP models, retinal microglia exhibit marked activation accompanied by increased TLR4 expression and enhanced NF-κB signaling activity. Inhibition of this pathway suppresses TLR4 expression and reduces the transcription of downstream inflammatory mediators, including p50, IL-6, and TNF-α, suggesting that TLR4/NF-κB signaling contributes to microglia-mediated inflammatory amplification during glaucoma progression (38). Beyond microglia, TLR4/NF-κB signaling also participates in astrocyte-mediated neuroinflammatory responses. Reactive astrocytes, which are increasingly recognized as active contributors to glaucomatous neurodegeneration, can undergo NF-κB-dependent inflammatory activation following innate immune stimulation, leading to increased production of cytokines such as IL-1β, IL-6, and TNF-α. These inflammatory mediators may further impair retinal homeostasis and promote RGC injury (39). Moreover, adaptive immune components, particularly T cells, may interact with TLR4/NF-κB-associated glial activation. Infiltrating T cells can release inflammatory cytokines, including interferon gamma (IFN-γ) and interleukin-17 (IL-17), which enhance microglial and astrocytic activation and contribute to sustained neuroinflammatory responses. Therefore, TLR4/NF-κB signaling should be considered a multicellular inflammatory regulatory network rather than a microglia-specific pathway (40). The cytosolic DNA-sensing cGAS-STING pathway has been identified as a crucial driver of microglia-mediated neuroinflammation in glaucoma. In distinct murine models, dsDNA released from tissue injury activates microglial cGAS-STING signaling, which in turn induces deleterious macroglial (astrocyte and Müller cell) reactivity through cytokine-mediated interactions, progressively driving RGC apoptosis (24). Global or microglial-specific deletion of STING markedly alleviates glaucoma symptoms and protects against RGC degeneration and vision loss (24). Additionally, the cyclic GMP-AMP synthase (cGAS) antagonist A151 can inhibit this pathway, reduce neuroinflammation, ameliorate RGC death, and promote a shift in microglial polarization from a pro-inflammatory state toward a more anti-inflammatory-like phenotype in a retinal ischemia/reperfusion injury model (41). Beyond DNA-sensing pathways, inflammasome activation represents another major innate immune mechanism linking cellular stress to chronic neuroinflammation in glaucoma. The NLRP3 inflammasome consists of the NLRP3 sensor, the adaptor protein apoptosis-associated speck-like protein containing a CARD (ASC), and pro-caspase-1. Upon activation, it promotes caspase-1 cleavage and the maturation of pro-inflammatory cytokines, particularly IL-1β and IL-18 (42). In glaucoma, disease-associated stressors, including elevated IOP, oxidative stress, mitochondrial dysfunction, and DAMPs, may activate NLRP3 signaling in retinal microglia and other glial populations. NLRP3 activation enhances inflammatory cytokine release and maintains reactive microglial states, thereby contributing to a sustained neuroinflammatory environment and RGC degeneration (27, 43, 44). Experimental studies suggest that pharmacological inhibition of NLRP3 signaling can reduce neuroinflammation and protect RGCs in glaucoma-related models, although further investigation is required to determine its specificity, safety, and translational feasibility (27, 43, 44). Another key regulator is thioredoxin-interacting protein (TXNIP), which is significantly upregulated in the glaucomatous retina and pressure-stimulated microglia. TXNIP facilitates M1-like microglial polarization, and its ablation promotes RGC survival and alleviates visual function impairment. Mechanistically, TXNIP deletion promotes PI3K phosphorylation, and inhibition of PI3K suppresses the M1-like transformation tendency in microglia, indicating the involvement of the phosphoinositide 3-kinase/protein kinase B (PI3K/Akt) pathway. IL-17A also acts synergistically with TXNIP in regulating microglial polarization (45). Collectively, these findings illustrate that multiple interconnected innate immune pathways, including TLR4/NF-κB, cGAS-STING, NLRP3 inflammasome activation, and TXNIP-associated inflammatory regulation, coordinate immune and glial responses in glaucoma. Complement signaling also represents a major innate immune mechanism closely associated with microglial activation, synaptic remodeling, RGC vulnerability, and optic nerve degeneration. Its role is discussed in greater detail in the following section.

### 2.3 Complement pathway activation in glaucomatous neurodegeneration

The complement system is a major component of innate immunity and has increasingly been implicated in glaucomatous neurodegeneration. Under physiological conditions, complement signaling contributes to immune surveillance, debris clearance, and synaptic refinement (46–48). However, excessive or persistent complement activation may promote maladaptive neuroinflammation, aberrant synaptic pruning, and neuronal injury (25, 40, 49, 50). In glaucoma, increased expression or deposition of complement components in the retina and optic nerve head suggests that complement dysregulation may contribute to RGC injury and degeneration (49).

One important mechanism through which complement may contribute to glaucomatous neurodegeneration is synaptic and dendritic remodeling. In an experimental glaucoma model, inhibition of the classical complement pathway prevented early dendritic and synaptic degeneration, supporting the involvement of complement signaling in the early stages of glaucomatous neurodegeneration (25). These findings suggest that complement-mediated synaptic and dendritic injury may represent an early pathogenic process rather than merely a secondary response to overt RGC loss.

Among complement-related pathways, the C3a–C3aR1 axis appears particularly relevant. Genetic disruption of C3a receptor 1 (C3ar1) in the DBA/2J mouse model of ocular hypertension reduces the risk of optic nerve degeneration without affecting IOP elevation (40). Microglia and other myeloid-derived cells express C3ar1, and C3ar1 deficiency alters microglial gene expression, particularly pathways associated with neuroinflammation (40). Network analysis further indicates that IL-10 signaling is linked to C3aR1-associated microglial regulation, suggesting that C3aR1 may influence the balance between inflammatory and regulatory microglial states (40). These findings support a pressure-independent role for C3aR1 signaling in glaucomatous neurodegeneration.

Emerging evidence further suggests that C3aR1-associated inflammatory signaling may converge on the N4BP1–JAK2/STAT3 axis. In experimental glaucoma, upregulation of N4BP1 has been linked to activation of JAK2/STAT3 signaling and pyroptosis-related inflammatory injury, thereby potentially amplifying retinal neuroinflammation and RGC damage (51). Although the precise cell-type-specific roles of this pathway remain to be fully defined, these findings suggest that JAK2/STAT3 signaling may function as a downstream inflammatory effector connecting complement-associated microglial activation with pyroptotic cell death. Further studies are needed to determine whether targeting this axis provides neuroprotection without disrupting the physiological immune and repair functions of JAK/STAT signaling.

Complement signaling may also interact with inflammatory and neurovascular pathways in glaucoma. Reactive astrocytes and Müller cells may produce complement components in response to stress or cytokine stimulation, thereby amplifying local complement activity (52, 53). Complement-derived fragments such as C3a and C5a can promote glial activation, cytokine release, and immune -cell recruitment. In addition, blood-retinal barrier disruption may facilitate the entry of circulating complement proteins and inflammatory mediators into the retinal microenvironment, while local complement activation may aggravate endothelial injury and vascular inflammation. Thus, complement activation may participate in a feed-forward loop involving glial reactivity, neurovascular dysfunction, and chronic immune activation (52, 53).

The diverse physiological functions of complement highlight the importance of defining its temporal, spatial, and cell-type-specific actions in glaucoma. Future studies should determine whether complement activation represents an initiating event, an amplifier of existing neuroinflammation, or a secondary response to neuronal injury in different disease contexts. Overall, complement dysregulation may link synaptic remodeling, microglial inflammatory regulation, blood-retinal barrier dysfunction, and RGC degeneration through mechanisms that are not fully explained by IOP elevation (**Figure 1**).

**Figure 1:**
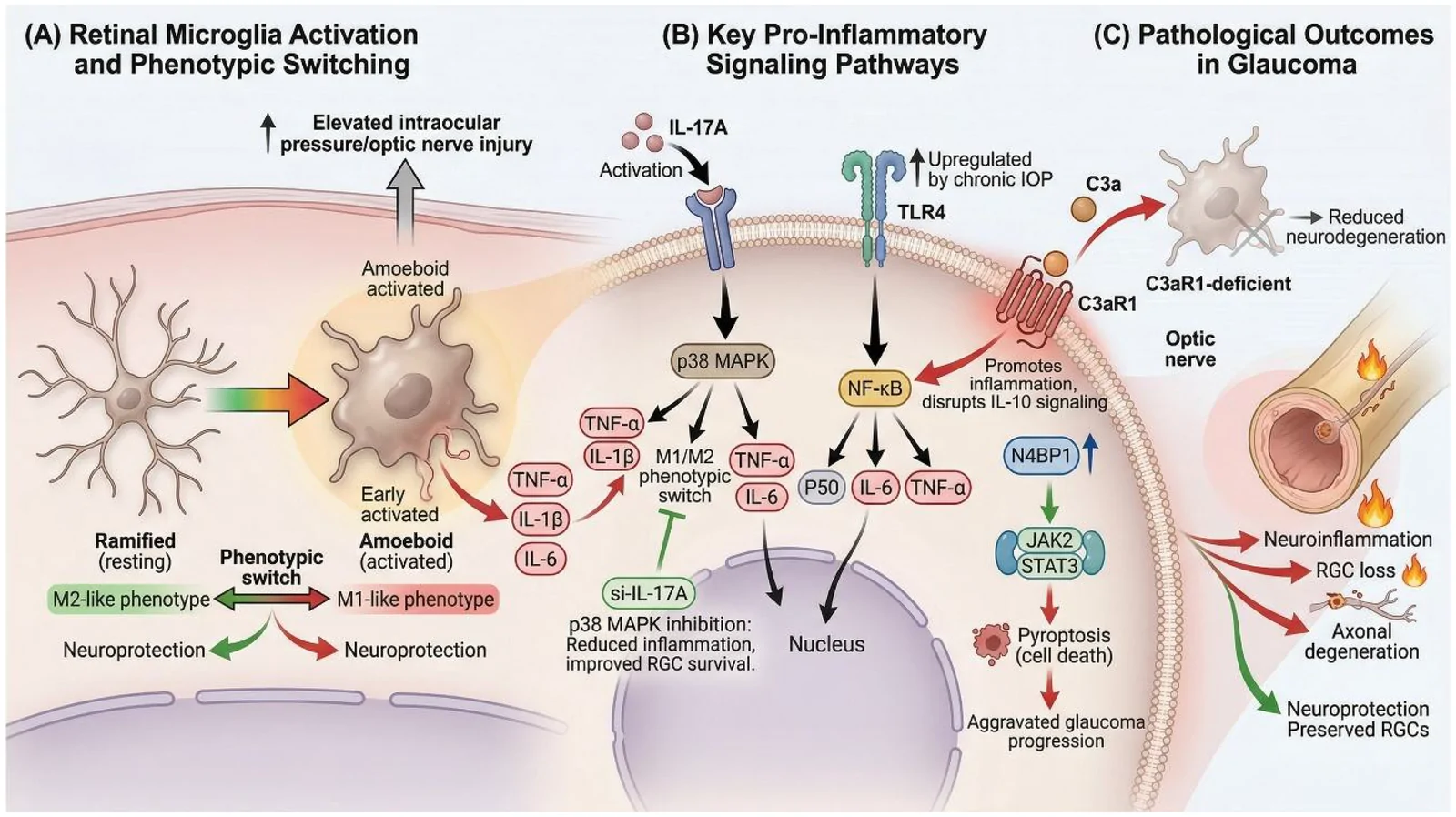
The core role of microglia-mediated innate immune responses in glaucoma. Schematic illustration of retinal microglial activation, phenotypic switching, inflammatory signaling, and pathological outcomes in glaucoma. **(A)** Elevated intraocular pressure and optic nerve injury induce the transition of retinal microglia from a ramified, resting state to an amoeboid, activated state. Activated microglia may acquire M1-like or M2-like features. Although M1-like features are generally associated with pro-inflammatory effects and M2-like features with potentially reparative responses, both states are context-dependent and neither is uniformly harmful or neuroprotective in glaucoma. **(B)** Key signaling pathways involved in microglial-mediated inflammation. IL-17A activates the p38 MAPK pathway, while chronic intraocular pressure upregulates TLR4 signaling. C3a binding to C3aR1 promotes inflammation and may disrupt IL-10-related signaling. Activation of p38 MAPK and NF-κB contributes to the production of pro-inflammatory mediators, including TNF-α, IL-1β, and IL-6, and promotes M1/M2 phenotypic switching. The N4BP1–JAK2/STAT3 axis is associated with pyroptosis and aggravated glaucoma progression. Inhibition of IL-17A by siIL-17A or inhibition of p38 MAPK reduces inflammatory responses and improves retinal ganglion cell survival. **(C)** Pathological consequences of microglial activation in glaucoma, including neuroinflammation, retinal ganglion cell loss, and axonal degeneration. C3aR1 deficiency is associated with reduced neurodegeneration, neuroprotection, and preservation of retinal ganglion cells.

## 3 Metabolic reprogramming and immune regulatory dysfunction in astrocytes

### 3.1 Astrocyte dysfunction and its association with neuroinflammation

In normal-tension glaucoma (NTG), which is characterized by progressive RGC loss and optic nerve damage despite the absence of elevated IOP, astrocyte dysfunction may contribute to IOP-independent mechanisms of optic neuropathy and represents a potential therapeutic target (54). Astrocytes are resident glial cells with essential metabolic, homeostatic, and immunoregulatory functions in the retina and optic nerve. Their dysfunction may increase axonal and neuronal vulnerability in glaucomatous neurodegeneration (54). This is supported by evidence from an experimental autoimmune glaucoma (EAG) model, which demonstrates that astrocyte metabolic reprogramming contributes to neurodegeneration (54). More broadly, in glaucoma pathogenesis, astrocytes are recognized as pivotal players, with their pathological metabolic reprogramming and heterogeneity profoundly impacting RGC survival (54, 55). Emerging perspectives redefine glaucoma as an astrocyte-driven neurodegeneration in the optic nerve head (ONH), where early IOP-induced remodeling of the ONH astrocyte network precedes RGC axonal damage, potentially due to impaired astrocytic regulation of electrophysiological homeostasis (56–58). The activation and reactivity of astrocytes are central to the neuroinflammatory process in glaucoma. Activated astrocytes exhibit morphological changes such as hypertrophy and increased expression of glial fibrillary acidic protein (GFAP), which is linked to glaucoma pathogenesis and plays a pivotal role in astrocyte-driven neuroinflammation (56–58). This reactive state, often termed astrogliosis, may become maladaptive when astrocytes lose homeostatic and neuroprotective functions and acquire pro-inflammatory or potentially neurotoxic properties (60, 61). In retinal injury models, neurotoxic reactive astrocytes have been shown to contribute to RGC death (61). Whether comparable astrocyte states are necessary or sufficient to drive RGC degeneration in chronic glaucoma remains to be determined (61). The molecular regulation of this neuroinflammation involves complex interactions. Astrocytes can be polarized into distinct phenotypes, such as the neurotoxic A1-like and neuroprotective A2-like states, influenced by cytokines like C1q, IL-1α, and TNF-α released from microglia and other immune cells (62). In glaucoma, ocular hypertension can trigger the production of these cytokines, driving the formation of neurotoxic A1-like astrocytes, and ablation of this pathway reduces RGC death (62). Furthermore, astrocytes are key regulators of the glutamate-glutamine metabolic cycle, essential for maintaining neuronal survival and inhibiting excitotoxicity (54). Dysfunction in this metabolic homeostasis, as seen in astrocyte metabolic reprogramming, can exacerbate neuronal vulnerability (55). Therefore, targeting astrocyte dysfunction and associated neuroinflammatory cascades may represent a promising adjunctive neuroprotective strategy for glaucoma, including IOP-independent mechanisms relevant to NTG (54, 59, 62, 63).

### 3.2 Regulation of astrocyte metabolism by the Ras-Raf-MEK/ERK signaling pathway

In the context of glaucomatous neurodegeneration, particularly within experimental models, astrocytes undergo significant metabolic reprogramming that disrupts crucial homeostatic functions. Studies using an EAG model have shown that undergo metabolic alterations. Modulation of the Ras–Raf–MEK/ERK signaling pathway can partially restore glutamate–glutamine metabolism in these cells (54). This pathway’s regulation is critically important because astrocytes are the key cellular mediators of the glutamate-glutamine cycle in the retina; their metabolic stability is vital for preventing excitotoxic neuronal damage and ensuring RGC survival (54). The intervention in this signaling cascade effectively alleviates immune-mediated inflammation in astrocytes, thereby creating a protective environment for RGCs against neuroinflammatory injury (54). Within this mechanistic framework, SynGAP1 has been proposed as a potential regulator of astrocyte metabolic reprogramming and Ras-related signaling in the EAG model (54). The broader significance of astrocyte metabolic function in glaucoma is underscored by studies showing that astrocyte heterogeneity and pathological metabolic shifts are central to RGC damage (54) (55). In a mouse model of elevated IOP, optic nerve astrocytes exhibited region-specific and temporally distinct transcriptional responses. ONH astrocytes showed increased mitochondrial biogenesis, ATP production, and a greater reliance on oxidative phosphorylation, supporting a role for metabolic adaptation during glial stress responses (64). Targeting specific metabolic pathways in astrocytes can yield neuroprotection. For example, the lipoxin pathway, which is functionally expressed in astrocytes across species, is dysregulated during ocular hypertension, leading to increased 5-lipoxygenase activity and astrocyte reactivity; treatment with lipoxin B4 (LXB4) can mitigate this reactivity and restore protective lipoxin generation (65). Vitamin C was reported to increase astrocytic secreted phosphoprotein 1 (SPP1) expression through E2F transcription factor 1 (E2F1)-associated mechanisms. It also promote astrocyte-associated neuroprotective functions, including neurotrophic-factor signaling and mitochondrial metabolic pathways (66). However, because oral vitamin C also reduced IOP in the microbead model, the relative contributions of IOP lowering and SPP1-mediated neuroprotection require further clarification. Specific ceramide species differentially affect RGCs and astrocytes. C16:0- and C18:1-ceramides contribute to RGC vulnerability, whereas C18:0-, C18:1-, and C24:0-ceramides increase TNF-α secretion from cultured human astrocytes. *In vivo*, ceramide synthase 5 (CerS5) deficiency reduced retinal C16:0 and C18:1 ceramides and protected RGC structure and function after ocular hypertension (67).For example, Piezo1-mediated mechanotransduction in ONH astrocytes can be activated by mechanical stretch and is associated with cytoskeletal redistribution and increased fibronectin expression, features of astrocyte reactivity (68). Furthermore, inhibition of mechanosensitive channels attenuates transforming growth factor beta 2 (TGFβ2)-induced actin cytoskeletal remodeling and reactivity in mouse ONH astrocytes (69). Together, these findings indicate that mechanical and biochemical stressors can converge on astrocyte reactivity, although the downstream signaling pathways linking these stimuli remain to be fully defined.

Therefore, the Ras-Raf-MEK/ERK axis serves as a critical regulatory node integrating metabolic, inflammatory, and mechanotransduction signals in astrocytes. Its modulation, potentially through targets like Syngap1, offers a strategic avenue to correct astrocyte metabolic dysfunction, suppress detrimental neuroinflammation, and ultimately protect RGCs, providing a promising direction for developing new therapies for glaucomatous and other optic neuropathies (54, 55, 68, 69) (**Figure 2**).

**Figure 2:**
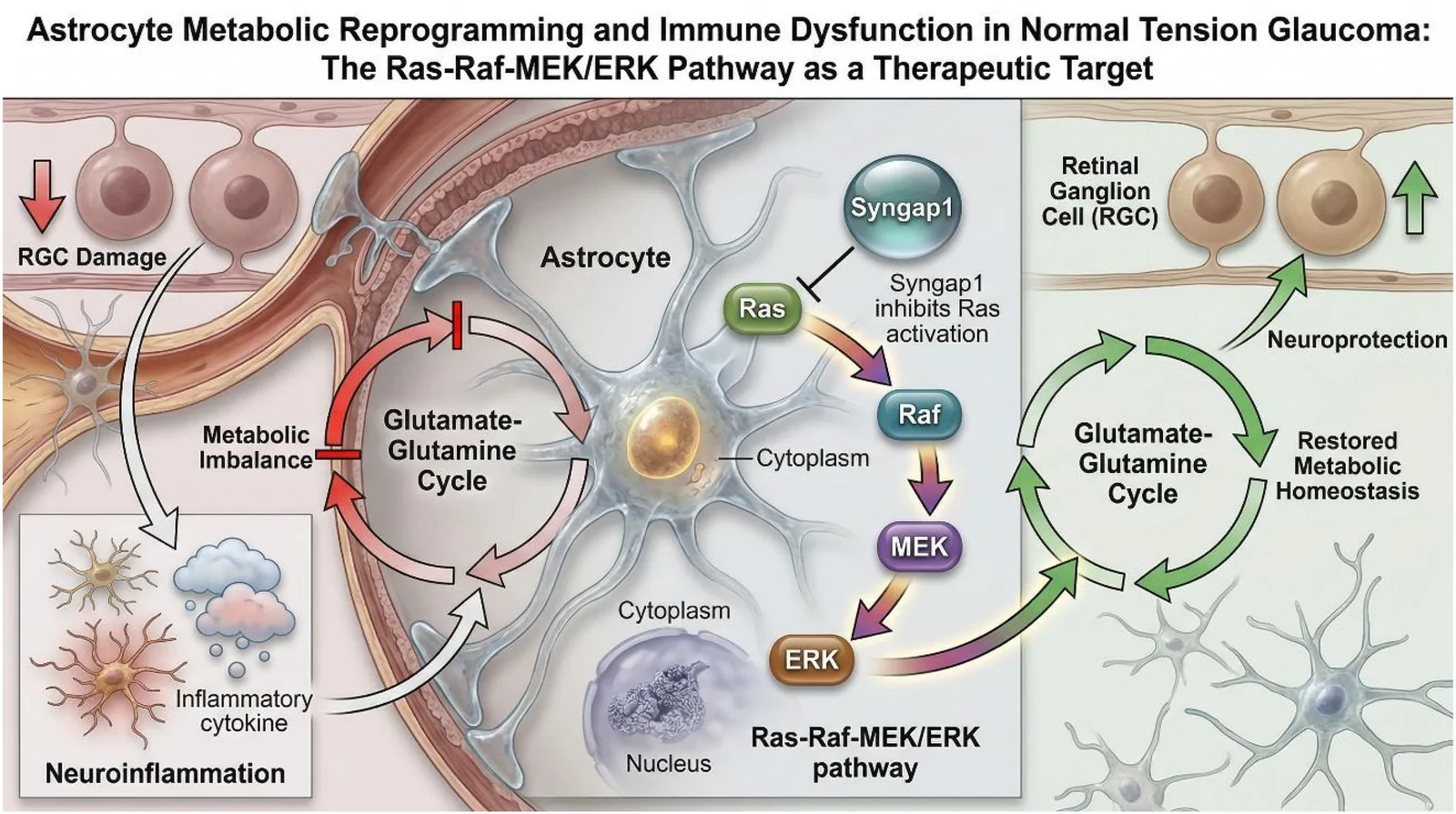
Metabolic reprogramming and immune regulatory dysfunction in astrocytes. Schematic illustration of astrocyte metabolic and immune dysregulation in normal-tension glaucoma. Retinal ganglion cell (RGC) damage and neuroinflammation, accompanied by the production of inflammatory cytokines, contribute to metabolic imbalance and disruption of the glutamate–glutamine cycle. Within astrocytes, Syngap1 inhibits Ras activation, thereby modulating the Ras–Raf–MEK/ERK signaling pathway. Restoration of this pathway is proposed to improve glutamate–glutamine cycling and re-establish metabolic homeostasis, ultimately reducing neuroinflammation, promoting neuroprotection, and preserving RGC integrity. Arrows indicate activation or regulatory progression, whereas blunt-ended lines indicate inhibition.

## 4 Involvement of adaptive immunity and T-cell receptor signaling pathways

### 4.1 T cell infiltration and its role in glaucoma pathology

Accumulating evidence underscores the involvement of the adaptive immune system, particularly T cells, in the chronic neurodegenerative process of glaucoma. T cells may gain access to the retina in association with endothelial activation and altered blood-retinal barrier trafficking. Clinical and experimental studies have provided evidence of T-cell involvement in glaucoma, although direct detection of T cells within human glaucomatous retinal tissue remains limited (70–72). For instance, CD4+ T cells from glaucoma patients exhibit enhanced activation and a bias toward T helper 1 (Th1) responses, which correlate with the degree of visual impairment (70). This infiltration is not merely correlative but functionally significant, as Th1 cells have been shown to directly target RGC and integrate into pro-inflammatory glial networks, thereby contributing to progressive RGC loss (70). The mechanism of T cell entry into the neural retina involves the upregulation of specific adhesion molecules on retinal vasculature. Th1 cells induce the expression of vascular cell adhesion molecule 1 (VCAM-1) on retinal microvessels, facilitating their transmigration (70). Furthermore, a distinct subset of gut-homing integrin β7+ CD4+ T cells has been implicated. In a mouse model of elevated intraocular pressure (EIOP)-triggered glaucoma, these β7+ CD4+ T cells infiltrate the retina during the progressive phase by eliciting the expression of mucosal vascular addressin cell adhesion molecule 1 (MAdCAM-1) on retinal endothelial cells (71). MAdCAM-1, minimally detected in healthy retinas, serves as a portal for T cell entry, and its neutralization with a specific antibody can ameliorate RGC loss and glial activity (71). This process is part of a broader “gut-retina” axis, where EIOP-induced β7+ CD4+ T cells first home to the gut, undergo transcriptional reprogramming associated with autoimmune and mucosal immunity pathways, and subsequently gain the competence to cross the BRB (71). The hypothesis that glaucoma may possess autoimmune features is further supported by experimental studies demonstrating HSP-related T-cell responses and HSP-dependent neurodegeneration (14) (73). Peripheral blood studies further suggest that T-cell dysregulation in glaucoma extends beyond the ocular microenvironment. Alterations in circulating CD4+ T cells and regulatory T cells (Tregs), including increased CD38 expression, have been reported in patients with primary open-angle glaucoma (POAG) and correlate with disease severity (74). Changes in Th1-, Th17-, and Treg-associated immune profiles have also been described, indicating an imbalance between pro-inflammatory and immunoregulatory responses (75, 76). These peripheral lymphocyte alterations, together with changes in circulating cytokines and other immune-related factors, may have potential as candidate biomarkers for disease stratification and progression monitoring when combined with conventional clinical parameters (75). However, peripheral immune profiles do not directly demonstrate T-cell infiltration into ocular tissues and may be influenced by demographic factors, systemic comorbidities, medication use, and disease stage. Therefore, larger longitudinal studies are needed to validate their clinical utility. Bioinformatic analyses of publicly available POAG transcriptomic datasets have also inferred changes in immune-cell-related signatures, including CD4+ T-cell- and Treg-associated signals (77). However, these computational estimates require experimental validation and should not be interpreted as direct evidence of ocular T-cell infiltration. Collectively, these findings establish that T-cell-related immune dysregulation in glaucoma involves both local pathogenic events within the retina and systemic alterations detectable in peripheral blood. T-cell infiltration, driven by specific adhesion pathways and potentially primed by systemic or commensal antigens, may exacerbate RGC death and visual dysfunction, while peripheral immune signatures may provide complementary information for future biomarker development.

### 4.2 CD3 zeta chain–associated signaling in experimental RGC injury and its potential neuroprotective relevance

CD3ζ-associated signaling has been implicated in experimental RGC degeneration; however, direct evidence linking this pathway to chronic glaucomatous neurodegeneration remains limited. In a mouse optic nerve crush (ONC) model, which reproduces axonal injury and RGC loss but does not fully recapitulate the chronic pressure-related pathophysiology of glaucoma, intravitreal adeno-associated virus serotype 9 (AAV9)-mediated CD3ζ knockdown improved RGC survival (78). In this study, CD3ζ, lymphocyte-specific protein tyrosine kinase (LCK), and zeta-chain-associated protein kinase 70 (ZAP70) were detected in RGCs and were upregulated after ONC injury, indicating that these TCR-associated molecules may have noncanonical or cell-intrinsic roles in injured RGCs (78). CD3ζ knockdown was associated with recovery of p38 and ERK1/2 phosphorylation and reduced phosphorylation of the NF-κB p65 subunit in retinal tissue after ONC (78). It also reduced retinal mRNA expression of TNF-death-associated responses after ONC; however, the cell-specific mechanisms and causal relationship between CD3ζ-associated signaling, MAPK/NF-κB modulation, and RGC survival require further investigation.IL-17A has also been implicated in experimental glaucoma-related RGC injury. In chronic ocular hypertension and N-methyl-D-aspartate (NMDA)-induced RGC injury models, IL-17A aggravated RGC apoptosis, Müller cell gliosis, and inflammatory responses through a pathway involving IL-17 receptor-associated Act1/TRAF6/NF-κB signaling, whereas intravitreal IL-17A neutralization alleviated these pathological changes (79). However, IL-17A neutralization also reduced IOP in the chronic ocular hypertension model; therefore, the extent to which its neuroprotective effect reflects direct immunomodulation rather than IOP reduction remains unclear (79). Collectively, CD3ζ-associated signaling and IL-17A-related inflammatory pathways represent potential targets for experimental RGC protection. Nevertheless, validation in chronic ocular hypertension models, human glaucoma tissues, and longer-term studies is necessary before these approaches can be considered therapeutic strategies for glaucoma. Such interventions should currently be viewed as potential adjuncts to established IOP-lowering treatment (**Figure 3**).

**Figure 3:**
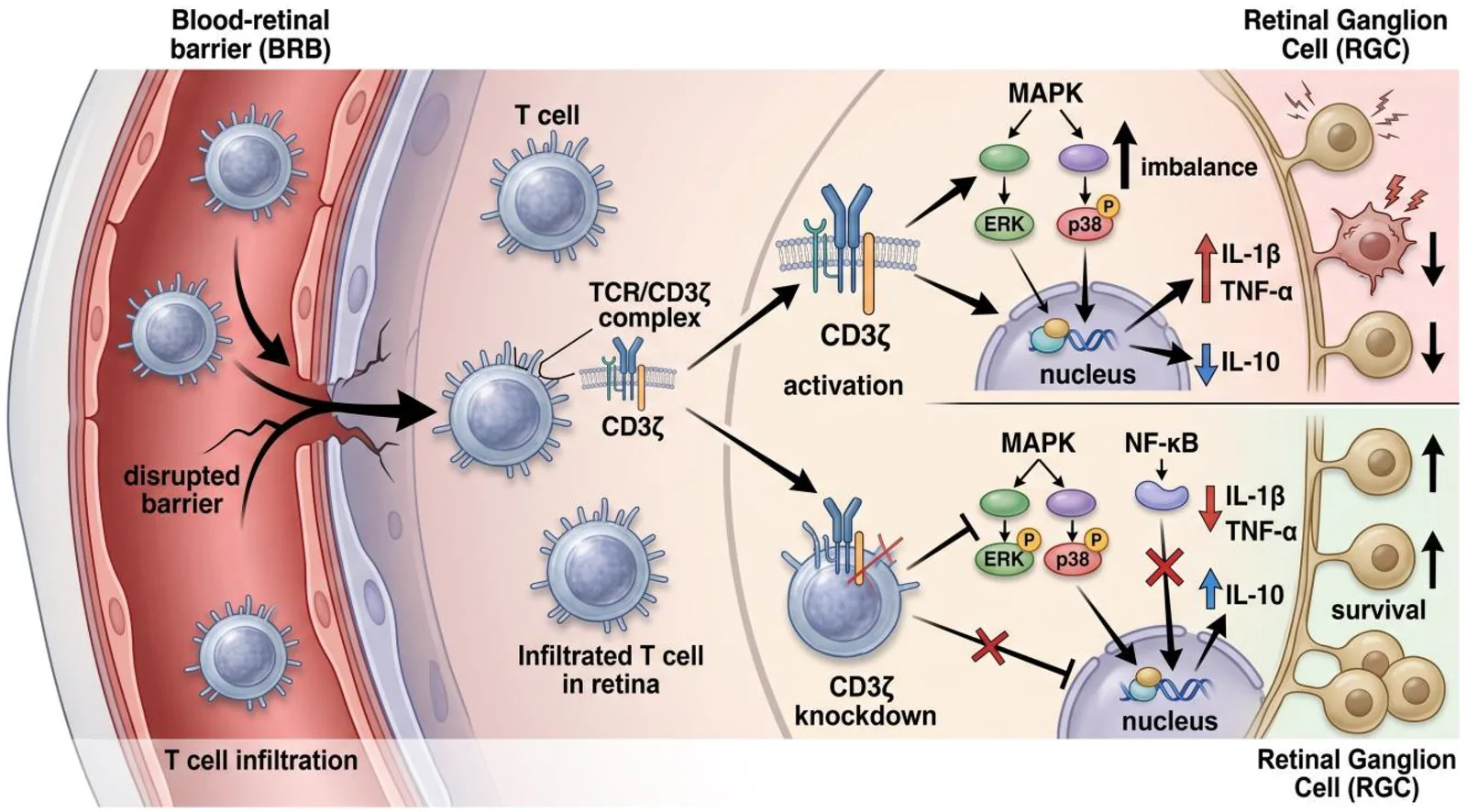
Proposed role of T-cell CD3ζ signaling in retinal inflammation and retinal ganglion cell injury following blood–retinal barrier disruption. Disruption of the blood–retinal barrier (BRB) facilitates T-cell infiltration into the retina. Activation of the T-cell receptor (TCR)/CD3ζ complex stimulates MAPK signaling, including the phosphorylation of ERK and p38, and promotes downstream inflammatory signaling. This response increases the production of the pro-inflammatory cytokines IL-1β and TNF-α while decreasing the anti-inflammatory cytokine IL-10, resulting in cytokine imbalance and retinal ganglion cell (RGC) injury. In contrast, CD3ζ knockdown suppresses MAPK and NF-κB signaling, reduces IL-1β and TNF-α levels, and increases IL-10 levels, thereby attenuating inflammation and promoting RGC survival.

## 5 Oxidative stress-immune inflammation interaction and neurovascular unit damage

### 5.1 NADPH Oxidase 2 as a bridge between oxidative stress and neuroinflammation

Pathological high IOP-induced oxidative stress is a critical mechanism in glaucoma, with NADPH oxidase 2 (NOX2) identified as a primary source of endothelial ROS. Evidence from a study investigating the interaction between oxidative stress and neuroinflammation in glaucoma demonstrates that NOX2-specific genetic deletion or pharmacological inhibition using gp91ds-tat effectively attenuates retinal oxidative stress and immune dysregulation following high IOP (80). This finding is corroborated by research showing that elevated IOP in mice impairs retinal arteriolar autoregulation and induces endothelial dysfunction, a process accompanied by increased ROS levels and upregulated NOX2 expression in the RGC layer and blood vessels (81). The central role of NOX2 in generating pathological oxidative stress is further highlighted in models of retinal ischemia–reperfusion (I/R) injury, in which I/R injury induced the upregulation of NOX2 components, and its suppression is associated with reduced oxidative damage and improved outcomes (82). Moreover, in a model of severe hypertension-induced ocular damage, increased NADPH oxidase activity and superoxide production, accompanied by transcriptional upregulation of its isoforms including NOX2, were found in the retina, linking oxidative stress directly to hypertensive eye disease pathology (83). The therapeutic potential of targeting NOX2 is underscored by the efficacy of its specific inhibitor, gp91ds-tat, which was shown to rescue glaucomatous RGC loss and optic nerve axon degeneration by attenuating inner blood-retinal barrier (iBRB) injury and neurovascular unit (NVU) dysfunction (80). Because BRB impairment may represent an early event in glaucomatous neurodegeneration, its mechanistic relevance is discussed in detail below. Similarly, the novel NOX2 inhibitor GLX7013170 demonstrated neuroprotective and anti-inflammatory effects in models of retinal excitotoxicity and early-stage diabetic retinopathy, reducing oxidative damage and microglial overactivation (84). The protective effect of resveratrol against I/R-induced RGC loss and endothelial dysfunction was also linked to its ability to reduce nitro-oxidative stress, possibly via the suppression of NOX2 upregulation (85). Furthermore, NOX2 emerges as a key regulator in pressure-induced RGC ferroptosis, where its deletion or inhibition by hydrogen sulfide (H2S) prevents RGC loss, positioning NOX2 as a critical node connecting oxidative stress to non-apoptotic cell death pathways in glaucoma (86). Collectively, these studies establish NOX2 as a pivotal enzyme bridging pathological IOP to oxidative stress and subsequent retinal damage, validating its inhibition as a potent strategy to alleviate oxidative burden and immune dysregulation in glaucomatous neurodegeneration.

### 5.2 The NOX2/ET-1/ERK1/2 axis drives pro-inflammatory microglial polarization

Mechanistically, NOX2-derived ROS activate a pro-inflammatory signaling cascade that critically involves the induction of endothelium-derived endothelin-1 (ET-1) overexpression, which in turn activates the ERK1/2 signaling pathway. This axis mediates the shift of microglial activation toward a pro-inflammatory phenotype, thereby triggering a neuroinflammatory outburst (80). The study elucidating this pathway found that NOX2 deletion or inhibition with gp91ds-tat attenuated high IOP-induced damage, and this protection was associated with the inhibition of the ET-1/ERK1/2-transduced shift in microglial activation toward the pro-inflammatory phenotype (80). This process is intrinsically linked to concurrent damage of the inner blood-retinal barrier (iBRB) and dysfunction of the neurovascular unit (NVU). The same research demonstrated that the NOX2/ET-1/ERK1/2 pathway controls reactive glial cell proliferation contributing to neuroinflammatory responses associated with blood-retinal barrier dysfunction, establishing a direct mechanistic link between oxidative stress, specific inflammatory signaling, and barrier integrity loss (80). The involvement of NOX2 in driving inflammatory microenvironments is supported by other models of retinal injury. In retinal I/R injury, the upregulation of NOX2 components was associated with NF-κB activation, a master regulator of inflammation, and suppression of this pathway in mice heterozygous for Ctr1 led to reduced microglial activation (82). Furthermore, in a model of severe hypertension, increased NADPH oxidase activity was accompanied by reactive gliosis, as reflected by GFAP upregulation in the retina, reinforcing the connection between NOX-derived oxidative stress and glial-driven inflammation (83). The activation of microglia and macrophages is a common feature in NOX2-associated pathologies, as inhibition of NOX2 by GLX7013170 limited the overactivation of microglia/macrophages in models of excitotoxicity and diabetic retinopathy (84). Targeting NOX2, therefore, presents a dual therapeutic advantage: it not only reduces the foundational oxidative stress but also improves the immune microenvironment by suppressing the ET-1/ERK1/2 axis. This inhibition disrupts the signal that promotes microglial polarization to the detrimental M1 state, which is a key source of sustained neuroinflammation and neuronal toxicity. By mitigating this specific inflammatory cascade, neuroprotection is achieved not merely through antioxidant effects but via a more comprehensive modulation of the neuroimmune interface. This provides a novel therapeutic rationale for glaucoma management, moving beyond pressure-lowering to directly address the oxidative-inflammatory nexus that drives progressive RGC loss and optic nerve degeneration.

### 5.3 Blood-retinal barrier disruption as an early event in glaucomatous neurodegeneration

The BRB, particularly the inner blood-retinal barrier (iBRB), is a key component of the retinal neurovascular unit and is essential for maintaining retinal homeostasis and immune privilege. The iBRB is mainly formed by retinal vascular endothelial cells connected by tight junctions and is supported by pericytes, Müller glia, astrocytic endfeet, and basement membrane structures (19, 87). Disruption of this barrier may expose retinal neurons and glial cells to circulating inflammatory mediators, complement proteins, plasma-derived factors, and immune cells, thereby promoting neuroinflammation and RGC injury (19, 80) (87, 88).

Emerging evidence suggests that BRB dysfunction may occur early in glaucoma and may contribute to subsequent neurodegeneration rather than simply reflecting secondary tissue damage (19, 87). Elevated IOP can impair retinal vascular autoregulation, induce endothelial dysfunction, and increase oxidative stress, which collectively compromise iBRB integrity. In this context, the NOX2-dependent oxidative-inflammatory cascade provides an important mechanistic link. NOX2-derived ROS promote endothelial injury and ET-1/ERK1/2-mediated signaling, leading to reactive gliosis, pro-inflammatory microglial activation, and barrier dysfunction (80, 81).

A related mechanism involving immune-cell trafficking has been described at the optic nerve head. In the DBA/2J mouse model of glaucoma, GlyCAM1 expression in the ocular vasculature was associated with reduced entry of monocyte-like immune cells into the optic nerve head. Conversely, Glycam1 deficiency increased monocyte-like cell entry and was accompanied by greater RGC and optic nerve damage (89). These findings suggest that vascular regulation of peripheral myeloid cell trafficking may influence glaucomatous neurodegeneration. However, because this study primarily examined monocyte-like cell entry into the optic nerve head, it should not be interpreted as direct evidence of iBRB disruption or monocyte infiltration into the neural retina (89).

Once BRB permeability is increased, circulating immune mediators and complement components may enter the retinal microenvironment and further activate microglia, astrocytes, and Müller cells. This creates a feed-forward loop in which oxidative stress induces barrier disruption, barrier disruption amplifies immune activation, and chronic neuroinflammation accelerates RGC loss and optic nerve degeneration (19, 80, 87, 88). Therefore, preserving BRB integrity may represent an important preclinical neuroprotective strategy in glaucoma, particularly in combination with approaches targeting NOX2-derived oxidative stress, complement activation, and glial inflammatory responses (19, 80, 81, 88) (**Figure 4**).

**Figure 4:**
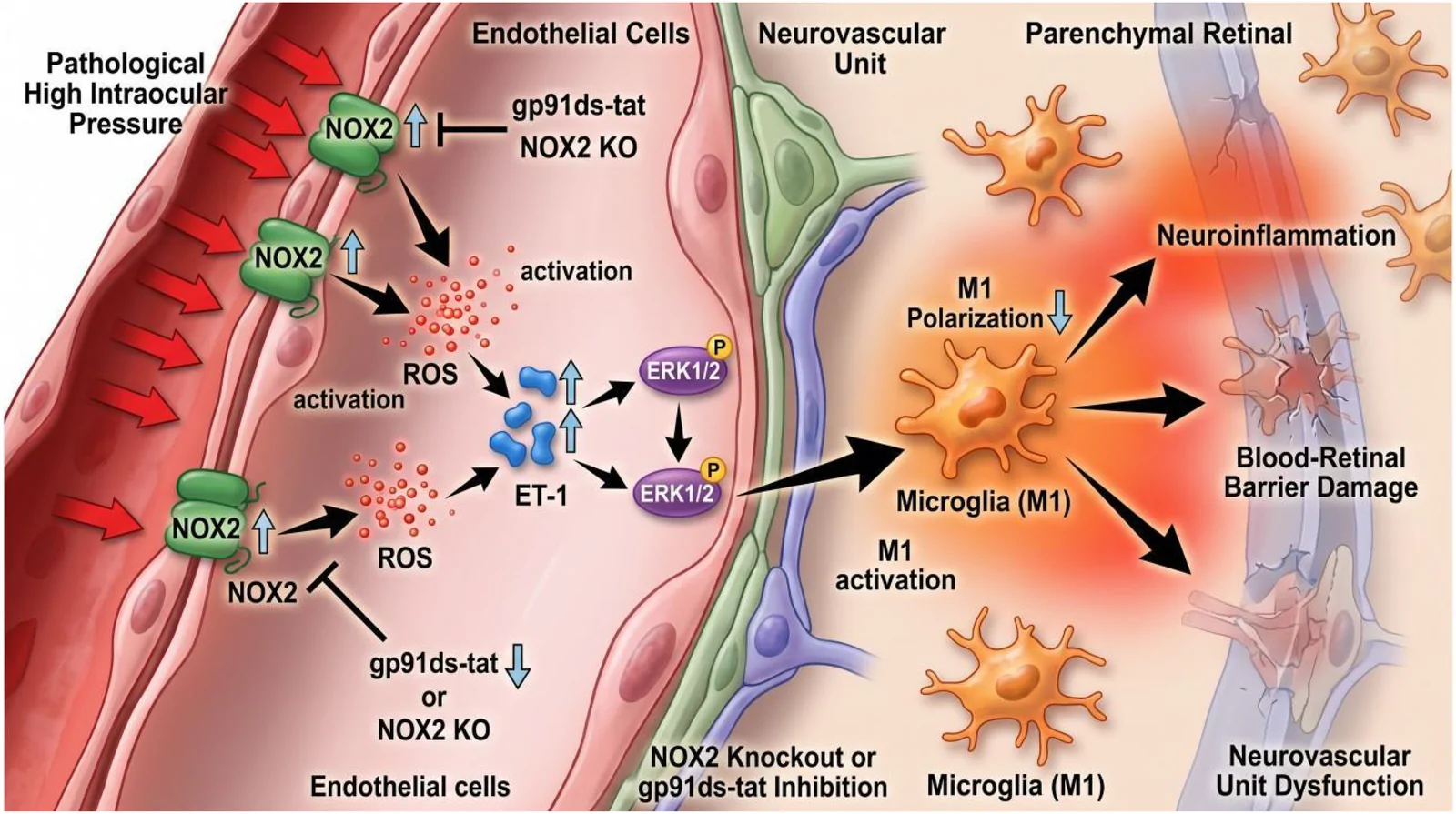
Proposed mechanism linking pathological intraocular pressure to blood–retinal barrier damage and neurovascular unit dysfunction. Pathological elevation of intraocular pressure activates NADPH oxidase 2 (NOX2) in retinal vascular endothelial cells, leading to increased production of reactive oxygen species (ROS) and upregulation of endothelin-1 (ET-1). The resulting activation of ERK1/2 signaling promotes microglial activation and M1 polarization in the retinal parenchyma. M1-polarized microglia contribute to neuroinflammation, blood–retinal barrier damage, and subsequent neurovascular unit dysfunction. NOX2 knockout (KO) or pharmacological inhibition with gp91ds-tat suppresses NOX2-mediated ROS production and attenuates the downstream pathological responses.

## 6 Multi-target therapeutic strategies and translational prospects

### 6.1 Complement inhibition: the most clinically advanced immune-targeted strategy

Among immune-related mechanisms implicated in glaucoma, the complement pathway currently represents one of the most clinically actionable therapeutic targets. Building on the mechanistic evidence summarized above, complement inhibition has emerged as therefore emerged as a promising neuroprotective strategy. Experimental glaucoma models have shown increased expression or deposition of complement components, and genetic or pharmacological modulation of complement signaling can attenuate neuroinflammation and protect RGCs in selected preclinical settings (25, 52). These findings provide a strong biological rationale for exploring complement inhibition as a neuroprotective strategy beyond IOP lowering. The translational relevance of complement inhibition is strengthened by active drug development in ophthalmology. Intravitreal complement inhibitors targeting C3 or C5 have entered clinical use or late-stage clinical development for retinal diseases such as geographic atrophy secondary to age-related macular degeneration (90, 91). Although these agents were not developed for glaucoma, they provide important proof-of-concept that local complement modulation in the eye is feasible, clinically testable, and potentially compatible with repeated intravitreal administration. This distinguishes complement inhibition from many other immune-based strategies in glaucoma, which remain largely confined to experimental models or computational prediction. However, direct extrapolation from retinal degenerative diseases to glaucoma should be made cautiously. Glaucoma is a chronic optic neuropathy with a slow disease course, heterogeneous mechanisms, and strong dependence on disease stage and IOP-related stress. Therefore, key translational questions include which patients show complement-driven pathology, when treatment should be initiated, which complement node should be targeted, and how treatment response should be measured. Biomarkers of complement activation in aqueous humor, plasma, retinal tissue, or imaging-based inflammatory readouts may help identify patients most likely to benefit and confirm target engagement (92–94). At present, complement inhibition should be viewed as a promising adjunctive neuroprotective strategy rather than a replacement for IOP-lowering therapy. Safety and regulatory issues are also central to the development of complement-targeted therapy for glaucoma. Complement signaling contributes to immune surveillance, debris clearance, and tissue homeostasis; therefore, long-term inhibition may carry risks such as intraocular inflammation, infection, impaired repair responses, or other retinal adverse events. Repeated intravitreal injections also introduce procedural risks and treatment burden (90, 91). Because glaucoma patients may require therapy for many years, the acceptable risk-benefit threshold may be different from that in advanced retinal degeneration. Future clinical trials should therefore incorporate careful ocular safety monitoring, standardized structural and functional endpoints, and biomarker-based patient stratification.

### 6.2 Inflammasome inhibition and microglial modulation: emerging therapeutic opportunities

Inflammasome signaling, particularly the NLRP3 pathway, represents another important immune target in glaucoma. Oxidative stress, mitochondrial dysfunction, lysosomal damage, and damage-associated molecular patterns can activate NLRP3 inflammasomes, leading to caspase-1 activation and release of pro-inflammatory cytokines such as IL-1β and IL-18. These events may promote maladaptive microglial activation, amplify neuroinflammation, and contribute to progressive RGC degeneration. Experimental studies suggest that inhibition of inflammasome signaling can reduce inflammatory injury and preserve RGCs in glaucoma-related models (95, 96).

Despite strong mechanistic support, inflammasome-targeted therapy remains less clinically mature than complement inhibition. Most evidence comes from preclinical systems, and glaucoma-specific clinical trials remain limited. A major challenge is that microglia and inflammasome pathways have both detrimental and protective roles depending on disease stage, local tissue context, and activation state. Broad immune suppression may therefore disrupt physiological immune surveillance or tissue repair (97). Future therapeutic development should focus on selective modulation of pathogenic microglial states rather than complete suppression of innate immune activity. Advanced single-cell transcriptomics, proteomics, and network-based analyses may help define disease-relevant immune cell states and identify druggable molecular nodes (98, 99). Previous bioinformatics and network pharmacology studies have nominated targets involved in inflammatory, metabolic, and stress-response pathways, including NF-κB, MAPK, STAT3, IL-6, IL-17, PIK3R6, FGF1, and selected GPCRs or metabolic enzymes (99, 100). These targets require further validation in human glaucoma tissues and clinically relevant models before they can be considered actionable therapeutic candidates. Nevertheless, such approaches provide a useful framework for moving from broad anti-inflammatory therapy toward precision immunomodulation.

### 6.3 Metabolic modulation, natural products, and network pharmacology as adjunctive strategies

In addition to direct immune targeting, metabolic and antioxidant strategies may provide complementary neuroprotection in glaucoma. RGCs have high energetic demands and are particularly vulnerable to mitochondrial dysfunction, oxidative stress, and impaired axonal transport. Natural products, nutraceuticals, and dietary interventions may influence these processes by modulating oxidative stress, inflammatory signaling, vascular function, and mitochondrial homeostasis. For example, citicoline has been reported to exert neuroprotective and anti-inflammatory effects in experimental and clinical contexts by stabilizing cellular membranes, reducing glutamate excitotoxicity, limiting free radical production, and modulating inflammatory cytokines and microglial activity (101, 102). Traditional Chinese Medicine components and other natural compounds have also been investigated using network pharmacology. These analyses suggest that some compounds may act on multiple nodes involved in apoptosis, inflammation, immunity, and cellular stress responses, including AKT1, STAT3, IL-6, IL-17, p53, MAPK, and NF-κB-related pathways (100). Dietary patterns such as the Mediterranean diet, which is rich in vitamins, carotenoids, flavonoids, and omega-3 fatty acids, have been proposed to support ocular health through anti-inflammatory and antioxidant mechanisms; however, direct evidence supporting their efficacy in glaucoma remains insufficient (103, 104). These strategies are attractive because glaucoma is multifactorial and may benefit from interventions that enhance neuronal resilience rather than target a single pathway. However, these approaches should be interpreted cautiously from a translational perspective. Many natural product and network pharmacology findings are hypothesis-generating rather than clinically validated. Limitations include uncertain ocular bioavailability, variable formulations, inconsistent dosing, potential herb–drug interactions, and insufficient glaucoma-specific randomized clinical evidence (101, 105). Therefore, natural products, nutraceuticals, and dietary strategies should currently be positioned as adjunctive or supportive approaches, not as substitutes for evidence-based IOP-lowering treatment or rigorously tested neuroprotective agents. Future studies should prioritize standardized formulations, pharmacokinetic characterization, clinically meaningful endpoints, and careful reference matching between claims and supporting evidence.

### 6.4 Biomarkers, delivery, safety, and clinical trial design

Translation of immune-based and neurovascular therapies for glaucoma requires reliable biomarkers for patient selection, target engagement, and treatment monitoring. Proteomic studies have identified candidate biomarkers linked to oxidative stress, inflammation, and complement dysregulation in aqueous humor (93, 106, 107). For example, reduced glutathione S-transferase P1 (GSTP1) expression in the aqueous humor of patients with primary open-angle glaucoma may reflect oxidative stress dysregulation and warrants further evaluation as a biomarker or therapeutic target (106). Complement-related proteins, cytokine signatures, inflammasome-related mediators, microglial activation markers, metabolic-stress indicators, and blood-retinal barrier-related readouts may further help define molecular glaucoma subtypes; however, their analytical validity and clinical utility remain to be established (93, 108, 109). Blood-based biomarkers may provide a more practical and minimally invasive approach for population-level screening, patient stratification, and longitudinal monitoring. Altered circulating immune-cell profiles, inflammatory cytokines, complement-associated factors, oxidative-stress markers, and metabolic signatures have been reported in glaucoma and may complement ocular imaging and aqueous humor analyses (75, 110–112). Stool-based biomarkers, particularly gut microbiota composition and microbially derived metabolites, may also be relevant given emerging evidence for a gut–retina immune axis in glaucoma (71, 113). However, blood- and stool-based signatures are susceptible to confounding by age, systemic inflammation, metabolic disease, medication use, diet, and technical variation. Therefore, their value as glaucoma biomarkers requires validation in large, longitudinal, and clinically well-phenotyped cohorts. Drug delivery remains a major challenge. Local ocular administration may enhance target-tissue exposure while limiting systemic exposure, but glaucoma requires durable, safe, and patient-acceptable treatment strategies. Repeated intravitreal injection, sustained-release formulations, periocular delivery, and gene-based approaches each offer potential benefits and risks (114). Emerging platforms, including nanodelivery systems and molecular imaging, should be discussed cautiously and supported by glaucoma-specific or closely relevant ocular evidence. Clinical trials of neuroprotective and immunomodulatory therapies are challenging because glaucoma progression is often slow and both structural and functional measures have inherent variability. Trials should use appropriate patient selection, sufficient follow-up, standardized optical coherence tomography and visual-field endpoints, and molecular biomarkers where feasible (115). Background IOP-lowering treatment, disease stage, progression rate, and baseline inflammatory or complement activity should also be considered. For complement-targeted or other immune-modulating therapies, safety monitoring should include intraocular inflammation, infection, retinal vascular events, systemic immune effects where relevant, and complications associated with repeated ocular procedures (116, 117). Overall, immune-based and multi-target therapies may extend glaucoma management beyond pressure control (**Table 1**). Complement inhibition currently has the strongest translational basis among immune-targeted approaches because complement inhibitors have entered late-stage clinical development and routine ophthalmic use for geographic atrophy secondary to age-related macular degeneration, although this experience cannot be assumed to translate directly to glaucoma (116–118). Inflammasome inhibition, microglial modulation, metabolic interventions, natural products, and strategies preserving neurovascular and blood-retinal barrier integrity remain less clinically mature. These approaches should be considered adjuncts to, rather than replacements for, established IOP-lowering therapy.

**Table 1:** Immune- and inflammation-related targets and pathways in glaucoma.

Immune target/pathway	Main role in glaucoma	Supporting evidence	Translational stage	Major limitation
TLR4/NF-κB	Promotes microglial and astrocytic activation and pro-inflammatory cytokine production	Pharmacological/preclinical; limited human observational evidence	Preclinical	Broad inflammatory functions and limited cell specificity
cGAS-STING	Links injury-associated DNA sensing to microglial activation and RGC degeneration	Genetic and pharmacological/preclinical evidence	Preclinical, with relatively strong mechanistic support	Chronic glaucoma relevance and retinal delivery require validation
NLRP3 inflammasome	Couples oxidative stress and mitochondrial dysfunction to IL-1β/IL-18-mediated inflammation	Pharmacological/preclinical evidence; limited genetic evidence	Emerging preclinical target	Possible protective roles of inflammasome signaling and inhibitor specificity
Complement pathway/C3aR1	Contributes to synaptic pruning, dendritic degeneration, microglial activation, and optic nerve injury	Genetic and pharmacological/preclinical evidence; human pathological/observational evidence	Preclinical glaucoma target with ophthalmic clinical precedent in other retinal diseases	Optimal target, treatment window, and long-term safety remain uncertain
N4BP1-JAK2/STAT3	Regulates inflammatory microglial activation and pyroptosis-related retinal injury	Genetic/mechanistic and pharmacological evidence, if supported by the cited study	Early preclinical	Evidence remains model-dependent and requires validation in chronic glaucoma
T-cell/TCR signaling, including CD3ζ	May sustain neuroinflammation through T-cell infiltration and cytokine release	Genetic/mechanistic and limited pharmacological evidence; human observational evidence	Emerging translational area	Pathogenic versus protective T-cell subsets remain unresolved
NOX2/ROS-ET-1/ERK1/2 axis	Connects oxidative stress, neurovascular dysfunction, and pro-inflammatory microglial activation	Pharmacological/preclinical evidence; limited human biomarker evidence	Preclinical	Broad biological functions and potential off-target effects
Ras-Raf-MEK/ERK signaling in astrocytes	Regulates astrocytic metabolic responses and inflammatory reactivity	Mechanistic/preclinical evidence	Early preclinical	Astrocyte-specific therapeutic relevance remains to be established

## 7 Conclusion

The intricate interplay between the innate and adaptive immune systems within the ocular microenvironment has emerged as a central paradigm in understanding the pathogenesis and progression of glaucomatous neurodegeneration. This review has synthesized current evidence positioning intraocular immunity, particularly the microglia- and astrocyte-mediated innate responses, at the heart of the degenerative cascade. These responses are shaped by a complex and context-dependent network of signaling pathways, including TLR4/NF-κB, MAPK, complement signaling, and, based on emerging experimental evidence, the N4BP1–JAK2/STAT3 axis. Their activation initiates a sustained inflammatory milieu that compromises neuronal survival. Notably, the involvement of adaptive immunity, specifically T cells and their TCR signaling pathways such as CD3ζ, adds a layer of sophistication to this model, revealing glaucoma not merely as a disease of isolated pressure-induced insult but as a disorder with significant immune-mediated components. This expanded view directly opens new therapeutic avenues focused on immune modulation, moving beyond the sole reliance on IOP reduction.

A critical insight from recent research is the profound crosstalk between oxidative stress and neuroinflammation, forming a vicious cycle that accelerates damage. Pathways such as NOX2/ROS generation and ET-1/ERK1/2-driven pro-inflammatory microglial activation are not isolated phenomena; they synergistically exacerbate one another. This interaction is a key driver behind the breakdown of the blood-retinal barrier and the subsequent dysfunction of the neurovascular unit, creating a permissive environment for progressive RGC loss. Understanding this bidirectional relationship is crucial, as it suggests that therapeutic strategies targeting only one arm of this axis may be insufficient. These findings suggest that combined modulation of oxidative stress and inflammatory signaling may warrant further investigation as a potential approach to neuroprotection.

In response to this complex pathophysiology, therapeutic strategies are evolving from a singular focus on IOP-lowering to a more nuanced, multi-target approach. Approaches that concurrently modulate neuroinflammation, oxidative stress, glial reactivity, and complement-associated injury may be particularly relevant to the multifactorial nature of glaucomatous neurodegeneration. Concurrently, the field is advancing toward precision targeting of specific molecular culprits, such as IL-17A, C3aR1, and Syngap1. These targets, identified primarily through mechanistic and preclinical studies, may provide a basis for developing more selective interventions; however, their therapeutic specificity, efficacy, and safety remain to be established. This dual strategy—broad-spectrum, multi-pathway modulation and high-precision molecular targeting—defines the new frontier in glaucoma neuroprotection, aiming to preserve visual function by directly safeguarding RGCs and their synaptic connections.

Looking forward, translating these promising concepts into clinical reality demands a concerted, interdisciplinary effort. The heterogeneity of glial and immune cell responses, even within seemingly uniform populations like microglia, necessitates a deeper, more granular understanding. Future studies should move beyond the simplified M1/M2 framework and use single-cell transcriptomics, spatial profiling, proteomics, and other multi-omics approaches to define glaucoma-specific microglial states, their temporal evolution, and their interactions with complement activation, oxidative stress, blood-retinal barrier dysfunction, and RGC degeneration. The integration of single-cell multi-omics technologies will be indispensable for mapping the precise phenotypic states and transcriptional profiles of these cells in different stages of glaucoma and in response to various therapies. Furthermore, computational modeling and systems biology approaches can help decipher the dynamic interactions within the vast immune signaling networks, predicting key nodes for therapeutic intervention and potential compensatory mechanisms that may arise.

Finally, overcoming the significant challenge of targeted delivery to the retina and optic nerve head remains a critical priority. Advances in nanotechnology, engineered viral vectors, and sustained-release implants are critical to ensure that therapeutic agents, whether small molecules, biologics, or gene therapies, reach their intended cellular targets at effective concentrations over prolonged periods. Although precise immunomodulation may ultimately contribute to slowing disease progression and preserving visual function, current evidence remains predominantly preclinical. The clinical feasibility, durability, safety, and neuroprotective efficacy of these approaches require validation in clinically relevant models and well-designed human studies. By fostering collaboration between immunologists, neuroscientists, bioengineers, and clinicians, the future of glaucoma management can shift from slowing progression to achieving genuine neurorestoration, fundamentally changing the prognosis for millions of patients worldwide.
